# High mean corpuscular volume as a predictor of esophageal cancer: A cohort study based on the Japanese Shizuoka Kokuho Database

**DOI:** 10.1371/journal.pone.0318791

**Published:** 2025-02-11

**Authors:** Shinsuke Sato, Emi Ohata, Eiji Nakatani, Philip Hawke, Hatoko Sasaki, Erina Nagai, Yusuke Taki, Masato Nishida, Masaya Watanabe, Ko Ohata, Hideyuki Kanemoto, Akira Sugawara

**Affiliations:** 1 Department of Gastroenterological Surgery, Shizuoka General Hospital, Shizuoka, Japan; 2 Graduate School of Public Health, Shizuoka Graduate University of Public Health, Shizuoka, Japan; 3 Department of Academic Services, 4DIN Ltd., Tokyo, Japan; 4 Department of Biostatistics and Health Data Science, Graduate School of Medical Science, Nagoya City University, Nagoya, Japan; 5 School of Pharmaceutical Sciences, University of Shizuoka, Shizuoka, Japan; The First Hospital of Jilin University, CHINA

## Abstract

Mean corpuscular volume (MCV) is known to increase with alcohol and tobacco consumption, and is therefore a potential predictive marker for esophageal cancer onset. However, this potential has not previously been examined using a large database. This study aims to clarify whether MCV is a predictor of esophageal cancer onset using health checkup data from a comprehensive health insurance claims database of a major administrative district in Japan. Health checkup data for 582,342 individuals recorded between April 2012 and September 2020 in the Shizuoka Kokuho Database were analyzed. Risk factors were assessed using both univariable and multivariable Cox proportional hazards models. Within the cohort, 1,562 health checkup participants (0.27%) had been diagnosed with esophageal cancer during the study period. Multivariable analysis revealed that risk of esophageal cancer onset was predicted by hypertension, smoking, systolic blood pressure, alcohol consumption, alcohol use disorder, body mass index, low-density lipoprotein cholesterol, and MCV. The cutoff value of MCV for predicting esophageal cancer onset was 104.086 fl. These results suggest that it may be appropriate to carry out endoscopy to detect esophageal cancer when MCV, a well-known indicator of alcohol and tobacco consumption, is greater than 104 fl.

## Introduction

Esophageal cancer is well known for its poor prognosis, resulting in more than 540,000 deaths worldwide in 2020 [1]. Eighty-five percent (51,2500) of cases were squamous cell carcinoma (SCC), and 14% (85,700) were adenocarcinoma. The incidence of esophageal SCC is particularly high in East Asia, including Japan, China, and Central Asia [2]. Drinking alcohol and smoking are well known to be strong risk factors for the development of esophageal SCC [3]. Smoking is also associated with a 2-3 times higher risk of developing adenocarcinoma, the most common histological type in the West, compared to nonsmokers [4,5].

Mean corpuscular volume (MCV) represents the average size of the red blood cells in a blood vessel. It is calculated by multiplying the hematocrit value by 10, then dividing by the number of red blood cells. It has long been known that heavy drinking and smoking increase MCV [6,7]. Thus, increased MCV is a potential predictive marker for the development of esophageal cancer. It has been reported that increased MCV is a risk factor for esophageal squamous cell carcinoma in both individuals with alcohol use disorder and those without [8–11]. However, as these previous reports were all case-control or single-center studies, their findings may have been subject to selection or information bias. Therefore, we carried out a more reliable retrospective cohort study using a large Japanese database to evaluate whether increased MCV is a predictive marker for the onset of esophageal cancer.

## Materials and methods

### Data Source

Shizuoka Prefecture is located in central Japan, with a population of approximately 3.6 million. This retrospective cohort study was conducted using the Shizuoka Kokuho Database (SKDB), an administrative claims database for beneficiaries of the two municipal government insurance systems provided by Shizuoka Prefecture: the National Health Insurance (NHI) system for individuals < 75 years of age, and the Late-Stage Elderly Medical Care System (LSEMCS) for those ≥ 75 years of age. In addition to basic subscriber information (sex, age, postal code, observation period, and reason for withdrawal including death), the data set includes the codes of the 10th revision of the International Statistical Classification of Diseases and Related Health Problems (ICD-10), as well as disease names, drug prescriptions, medical receipts, blood tests, and information on level of care. The SKDB also includes data from voluntary health checkups conducted annually at designated community centers and medical institutions for individuals aged 40 and older as part of the NHI and LSEMCS programs. The utility of the SKDB in assessing risk factors of disease onset has been demonstrated in several previous studies [12–14].

In this study, data on all registered individuals was preprocessed, including thorough cleaning and anonymization [15]. The data were accessed for research purposes in September 2022. We did not have access to any information that could identify individual participants during or after data collection. The study followed the Reporting of Studies Conducted using Observational Routinely-collected Health Data (RECORD) reporting guidelines [16].

### Study design and population

**Fig 1:**
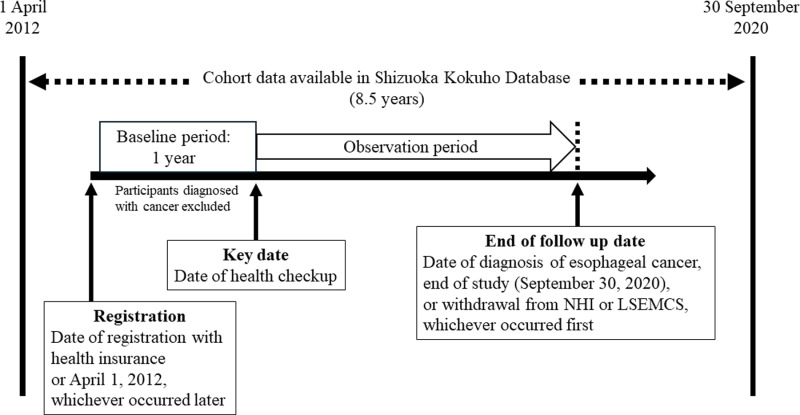
Study schema. Cohort entry was defined as a patient’s date of registration with health insurance or April 1, 2012, whichever occurred later. The key date was the initial date of an annual health checkup with > 12 months of continuous subscribership to the health insurance system. The baseline was the 12 months preceding the key date. The observation period was the interval between the key date and (1) the end of the study (September 30, 2020) or (2) the date of withdrawal from the health insurance system, whichever occurred first. NHI, National Health Insurance; LSEMCS, Late-Stage Elderly Medical Care System.

This retrospective cohort study was based on SKDB entries covering April 2012 to September 2020. The study design is shown in Fig 1. The key date was the initial date of an annual health checkup with > 12 months of continuous subscribership to the health insurance system. Those diagnosed with other cancers in the previous year (baseline period) were excluded.

### Outcome and covariates

Outcome was defined as time to occurrence of esophageal cancer. Occurrence was identified using ICD-10 code C15.

The covariates studied were age, sex, body mass index (BMI), current smoking, frequency of alcohol consumption, amount of alcohol consumption, comorbidities (including diabetes, hypertension, chronic lung disease, cerebrovascular disease, liver disease, myocardial infarction, arrhythmia, peripheral vascular disease, renal disease, and psychiatric disease), and blood laboratory values (including HbA1c and estimated glomerular filtration rate). Comorbidity variables were identified in the claims data by ICD-10 code. The search period was one year before the date of a health checkup. The comorbidity indexes utilized were the widely-used Charlson and Elixhauser indexes [17,18]. Current smoking, alcohol consumption, and physical activity were examined based on questionnaires taken during the annual health checkups.

### Statistical analysis

Continuous and categorical variables were summarized using means ±  standard deviation and frequencies (percentages). To compare baseline characteristics between patients who developed esophageal cancer and those who did not, a t-test and a chi-square test were used for continuous and categorical variables, respectively.

The Smirnov-Grubbs test was employed to identify and exclude outliers in the laboratory values. The test detects outliers by assuming that laboratory values follow a normal distribution. Each variable was individually tested only once, and values identified as outliers at a significance level of α =  0.05 were excluded from further analysis to ensure the robustness and reliability of the statistical results. To categorize MCV, we divided the MCV values into four groups by quartile, with Quartile 1 represented the lowest 25% of MCV values and Quartile 4 the highest 25%.

Univariable and multivariable Cox proportional hazards regression analysis was performed to explore predictors of esophageal cancer development. Hazard ratios (HRs), 95% confidence intervals (CIs) based on the Wald test, and corresponding P values were calculated. To generate a cumulative incidence curve verifying the proportional hazards assumption, event-free time was defined as the duration from the key date to the occurrence of esophageal cancer, with death or the end of follow-up treated as the censoring date. Variables included in the univariable model were sex, age, smoking, the comorbidities included in the Charlson and Elixhauser comorbidity indexes, and laboratory values. Spearman’s rank correlation coefficient was used to check correlations between potential predictors, and variables with an absolute correlation coefficient of ≥ 0.4 were considered correlated. Among correlated variables, one variable was selected based on clinical importance. All potential independent predictors were entered into a multivariable model.

To generate a precise cumulative incidence curve, event-free time was defined as the duration from the key date to the occurrence of esophageal cancer. The end of follow-up was treated as a censoring event, while death was considered a competing risk. By accounting for death as a competing event, we were able to estimate the cumulative incidence of esophageal cancer more accurately.

MCV cutoff values were determined based on conditional inference tree analysis. First, the data were sequentially divided into two groups according to MCV values. Next, the two groups were compared using permutation tests, and the variable with the smallest P-value was selected as the node for the grouping. This method was repeated for each subgroup until all separations were no longer significant or the smallest node was reached [19].

Missing values were not imputed in all analyses. A two-sided P-value of < 0.001 was considered statistically significant due to the use of a large-scale database. All statistical analyses were carried out using R version 4.3.1 (The R Foundation for Statistical Computing, Vienna, Austria) and EZR version 1.61 (Saitama Medical Center, Jichi Medical University, Saitama, Japan), a graphical user interface for R [20].

### Ethics

All data from SKDB were fully anonymized to rigorously maintain participant confidentiality. Consequently, individual informed consent was not required for this study. The research protocol for this study was approved by the Ethics Review Committee of the Shizuoka Graduate University of Public Health on June 22, 2021 (SGUPH_2021_001_019). This study was conducted in accordance with the principles of the Declaration of Helsinki.

## Results

### Demographics of participants

**Fig 2:**
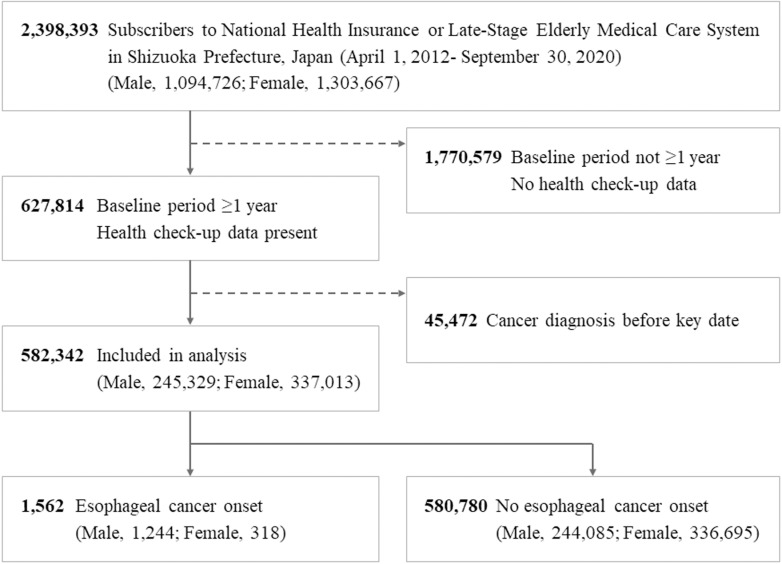
Flow diagram of cohort enrolment.

The analysis data set included 2,398,393 individuals. After eligibility was assessed, 1,816,051 individuals were excluded, leaving 582,342 participants to be included in the analysis. The median (longest) observation period was 2,156 (2,739) days. During the observation period, esophageal cancer had been diagnosed in 1,562 participants (0.27%) (Fig 2). The baseline characteristics of those diagnosed with esophageal cancer and those not diagnosed with the disease are shown in Table 1. Those diagnosed with esophageal cancer had higher MCV, mean corpuscular hemoglobin concentration (MCHC), and mean corpuscular hemoglobin (MCH) than the others.

**Table 1:** Baseline characteristics of participants.

Variable	Overall	With esophageal cancer	Without esophageal cancer	p-value
	n = 582342	n = 1,562	n = 580,780	
**Sex, n (%)**				**<0.001**
Male	245,329 (42%)	1,244 (80%)	244,085 (42%)	
Female	337,013 (58%)	318 (20%)	336,695 (58%)	
**Age, n (%)**				**<0.001**
Mean ± SD	67.9 ± 11.3	71.4 ± 8.13	67.9 ± 11.3	
< 40	4,720 (1%)	1 (0%)	4,719 (1%)	
40 to < 50	45,068 (8%)	16 (1%)	45,052 (8%)	
50 to < 60	52,754 (9%)	58 (4%)	52,696 (9%)	
60 to < 70	221,037 (38%)	604 (39%)	220,433 (38%)	
70 to < 80	173,228 (30%)	633 (41%)	172,595 (30%)	
80 to < 90	76,409 (13%)	232 (15%)	76,177 (13%)	
≥90	9,126 (2%)	18 (1%)	9,108 (2%)	
**Smoke, n (%)**	70,542 (12%)	393 (25%)	70,149 (12%)	**<0.001**
**Alcohol consumption, frequency, n (%)**				**<0.001**
Daily	102,399 (21%)	726 (55%)	101,673 (21%)	
Sometimes	99,160 (20%)	190 (14%)	98,970 (20%)	
Rarely	295,115 (59%)	397 (30%)	294,718 (59%)	
**Alcohol consumption, amount, n (%)**				**<0.001**
< 20g	273,572 (72%)	438 (38%)	273,134 (72%)	
20–40g	70,189 (18%)	379 (33%)	69,810 (18%)	
40–60g	28,309 (7%)	265 (23%)	28,044 (7%)	
≥ 60g	7,850 (2%)	60 (5%)	7,790 (2%)	
**Comorbidities, n (%)**				
Cerebrovascular disease	71,537 (12%)	224 (14%)	71,313 (12%)	0.013
Dementia	13,021 (2%)	28 (2%)	12,993 (2%)	0.235
Myocardial infarction	7,494 (1%)	30 (2%)	7,464 (1%)	0.026
Renal disease	10,360 (2%)	33 (2%)	10,327 (2%)	0.318
Congestive heart failure	41,568 (7%)	126 (8%)	41,442 (7%)	0.154
Peripheral vascular disease	46,455 (8%)	149 (10%)	46,306 (8%)	0.023
Chronic pulmonary disease	100,434 (17%)	277 (18%)	100,157 (17%)	0.61
Peptic ulcer disease	75,613 (13%)	222 (14%)	75,391 (13%)	0.148
Hemiplegia or paraplegia	3,071 (1%)	6 (0%)	3,065 (1%)	0.434
Diabetes	25,271 (4%)	82 (5%)	25,189 (4%)	0.077
Liver disease	70,169 (12%)	250 (16%)	69,919 (12%)	**<0.001**
Cardiac arrhythmias	54,831 (9%)	184 (12%)	54,647 (9%)	0.001
Valvular disease	17,449 (3%)	62 (4%)	17,387 (3%)	0.024
Pulmonary circulation disorders	819 (0%)	2 (0%)	817 (0%)	>0.999
Other neurological disorders	15,247 (3%)	46 (3%)	15,201 (3%)	0.418
Hypothyroidism	9,085 (2%)	21 (1%)	9,064 (2%)	0.491
Peptic ulcer disease excluding bleeding	74,527 (13%)	218 (14%)	74,309 (13%)	0.17
Rheumatoid arthritis/collagen vascular diseases	16,191 (3%)	32 (2%)	16,159 (3%)	0.078
Coagulopathy	2,950 (1%)	9 (1%)	2,941 (1%)	0.698
Obesity	1,884 (0%)	3 (0%)	1,881 (0%)	0.36
Weight loss	1,099 (0%)	4 (0%)	1,095 (0%)	0.547
Fluid and electrolyte disorders	35,347 (6%)	89 (6%)	35,258 (6%)	0.538
Blood loss anemia	440 (0%)	0 (0%)	440 (0%)	0.637
Deficiency anemia	24,175 (4%)	76 (5%)	24,099 (4%)	0.156
Alcohol use disorder	2,388 (0%)	34 (2%)	2,354 (0%)	**<0.001**
Drug abuse	117 (0%)	1 (0%)	116 (0%)	0.27
Depression	28,157 (5%)	55 (4%)	28,102 (5%)	0.015
Psychosis	9,499 (2%)	14 (1%)	9,485 (2%)	0.022
**Laboratory test value, mean (SD)**				
BMI	22.7 (3.4)	22.3 (3.1)	22.7 (3.4)	**<0.001**
SBP (mmHg)	129.4 (17.3)	132.8 (17.1)	129.4 (17.3)	**<0.001**
DBP (mmHg)	74.7 (11.1)	76.1 (11.2)	74.7 (11.1)	**<0.001**
RBC (/μL)	443.9 (46.1)	434.8 (48.9)	443.9 (46.1)	**<0.001**
Hematocrit (%)	41.7 (4.1)	42.2 (4.3)	41.7 (4.1)	**<0.001**
Hemoglobin (g/dL)	13.6 (1.5)	13.9 (1.5)	13.6 (1.5)	**<0.001**
γ-GTP (U/L)	28.0 (19.6)	37.5 (25.3)	28.0 (19.6)	**<0.001**
AST (U/L)	23.3 (6.7)	24.8 (7.6)	23.3 (6.7)	**<0.001**
ALT (U/L)	19.3 (9.4)	18.6 (9.2)	19.3 (9.4)	0.002
MCH (pg)	30.7 (1.8)	32.0 (2.3)	30.7 (1.8)	**<0.001**
MCHC (%)	32.6 (1.1)	32.9 (1.2)	32.6 (1.1)	**<0.001**
MCV (fL)	94.1 (5.1)	97.4 (6.5)	94.1 (5.1)	**<0.001**
MCV quartile, n (%)				**<0.001**
Q1 (67.5 to < 91.09)	115,901 (25%)	162 (14%)	115,739 (25%)	
Q2 (91.09 to < 94.05)	115,734 (25%)	191 (16%)	115,543 (25%)	
Q3 (94.05 to < 97.14)	115,812 (25%)	257 (22%)	115,555 (25%)	
Q4 (97.14 to <= 120.34)	115,559 (25%)	580 (49%)	114,979 (25%)	
Fasting blood glucose (mg/dL)	96.8 (14.9)	99.1 (15.8)	96.8 (14.9)	**<0.001**
HbA1c (%)	5.7 (0.5)	5.6 (0.5)	5.7 (0.5)	**<0.001**
LDL (mg/dL)	124.2 (31.2)	112.3 (31.2)	124.2 (31.1)	**<0.001**
HDL (mg/dL)	62.3 (16.5)	61.6 (17.4)	62.3 (16.5)	0.085
Triglyceride (mg/dL)	111.1 (59.6)	113.0 (62.7)	111.1 (59.6)	0.219
eGFR (mL/minutes)	69.5 (15.6)	68.9 (16.1)	69.5 (15.6)	0.162
Serum creatinine (mg/dL)	0.8 (0.2)	0.8 (0.2)	0.8 (0.2)	**<0.001**
Uric acid (mg/dL)	5.2 (1.3)	5.7 (1.4)	5.2 (1.3)	**<0.001**

BMI, body mass index; SBP, systolic blood pressure; DBP, diastolic blood pressure; RBC, red blood cell count; γ-GTP, γ-glutamyltranspeptidase; AST, aspartate aminotransferase; ALT, alanine transaminase; MCH, mean corpuscular hemoglobin; MCHC, mean corpuscular hemoglobin concentration; MCV, mean corpuscular volume; LDL, low density lipoprotein cholesterol; HDH, high density lipoprotein cholesterol; eGFR, estimated glomerular filtration rate; Q, quartile

### Predictors of esophageal cancer onset

Potential risk factors for the onset of esophageal cancer were evaluated using univariable and multivariable Cox regression analysis.

The univariable analysis showed a correlation between red blood cell count, hematocrit level, and hemoglobin level (r ≥ 0.4). As shown in Table 1, hemoglobin and hematocrit levels were elevated in participants diagnosed with esophageal cancer compared to the others, despite this same group also having a lower red blood cell count.

Three separate multivariable analyses were performed for each variable to clarify how these factors are related to the onset of esophageal cancer. Correlations were found between the following variables: 1) between sex, frequency of alcohol consumption, amount of alcohol consumed, serum creatinine, hemoglobin, hematocrit, and uric acid levels; 2) between systolic and diastolic blood pressure; 3) between HbA1c and fasting blood glucose; 4) between MCV and MCH; 5) between MCHC, MCH, and hemoglobin levels; and 6) between γ-glutamyl transpeptidase (γ-GTP) and hemoglobin levels (S1 Table). One clinically important variable was selected for the multivariable analyses of these correlated factors. All three multivariable analyses showed that MCV was an independent risk factor for developing esophageal cancer (Table 2).

**Table 2:** Univariable and multivariable Cox regression analysis of esophageal cancer onset.

Variable (reference)		Category or unit	Univariable model			Multivariable model with RBC			Multivariable model with hemoglobin			Multivariable model with hematocrit		
			HR	95% CI	P-value	HR	95% CI	P-value	HR	95% CI	P-value	HR	95% CI	P-value
**Sex** (Female)		Male	5.69	5.03–6.43	** < 0.001**	4.51	3.82–5.32	** < 0.001**	NA	NA	NA	NA	NA	NA
**Age** (>40 years)		40 to < 50 years	1.45	0.19–11.00	0.716									
		50 to < 60 years	3.84	0.53–27.80	0.182									
		60 to < 70 years	8.70	1.22–61.90	0.031									
		70 to < 80 years	11.20	1.58–79.70	0.016									
		80 to < 90 years	9.50	1.33–67.80	0.025									
		≥90 years	8.13	1.09–60.90	0.041									
**Smoking** (Absence)		Presence	2.71	2.42–3.04	** < 0.001**	1.57	1.36–1.83	** < 0.001**	1.58	1.33–1.87	** < 0.001**	1.60	1.34–1.92	** < 0.001**
**Alcohol consumption, frequency** (Rarely)		Sometimes	1.48	1.24–1.76	** < 0.001**	NA	NA	NA	NA	NA	NA	NA	NA	NA
		Daily	5.48	4.85–6.19	** < 0.001**	NA	NA	NA	NA	NA	NA	NA	NA	NA
**Alcohol consumption, amount** (>20g)		20 to < 40g	3.51	3.06–4.03	** < 0.001**	NA	NA	NA	2.84	2.37–3.39	** < 0.001**	2.58	2.14–3.12	** < 0.001**
		40 to < 60g	6.26	5.37–7.29	** < 0.001**	NA	NA	NA	4.20	3.42–5.18	** < 0.001**	3.74	2.99–4.69	** < 0.001**
		≥60g	4.15	4.15–7.11	** < 0.001**	NA	NA	NA	3.96	2.83–5.54	** < 0.001**	3.56	2.47–5.14	** < 0.001**
		**Comorbidity** (Absence)												
Cerebrovascular disease		Presence	1.18	1.02–1.36	0.022									
Dementia		Presence	0.93	0.64–1.35	0.707									
Myocardial infarction		Presence	1.59	1.11–2.29	0.012									
Renal disease		Presence	1.32	0.94–1.87	0.111									
Congestive heart failure		Presence	1.21	1.00–1.45	0.044									
Peripheral vascular disease		Presence	1.18	1.00–1.40	0.052									
Chronic pulmonary disease		Presence	1.04	0.92–1.19	0.536									
Rheumatic disease		Presence	0.72	0.48–1.06	0.097									
Peptic ulcer disease		Presence	1.06	0.92–1.22	0.434									
Liver disease		Presence	1.36	1.19–1.56	** < 0.001**	0.99	0.83–1.18	0.915	1.02	0.83–1.26	0.844	1.04	0.84–1.30	0.699
Diabetes	Presence	1.26	1.01–1.57	0.041										
Hemiplegia or paraplegia		Presence	0.75	0.34–1.68	0.485									
Cardiac arrhythmias		Presence	1.27	1.09–1.49	0.002									
Valvular disease		Presence	1.34	1.04–1.73	0.023									
Hypertension		Presence	1.47	1.33–1.62	** < 0.001**	1.32	1.16–1.51	** < 0.001**	1.35	1.15–1.57	** < 0.001**	1.34	1.14–1.57	** < 0.001**
Hypothyroidism		Presence	0.87	0.57–1.34	0.535									
Coagulopathy		Presence	1.22	0.63–2.34	0.560									
Obesity		Presence	0.61	0.20–1.89	0.391									
Weight loss		Presence	1.42	0.53–3.80	0.481									
Fluid and electrolytedisorders		Presence	0.95	0.76–1.17	0.611									
Deficiency anemia		Presence	1.24	0.99–1.57	0.064									
Alcohol use disorder		Presence	5.93	4.22–8.33	** < 0.001**	1.84	1.10–3.07	0.020	2.48	1.57–3.91	** < 0.001**	2.03	1.16–3.55	0.013
Drug abuse		Presence	3.90	0.55–27.70	0.173									
Depression		Presence	0.74	0.56–0.96	0.025									
Psychosis		Presence	0.62	0.36–1.04	0.070									
**Laboratory values**														
BMI		3.4	0.90	0.85–0.94	** < 0.001**	0.81	0.76–0.88	** < 0.001**	0.79	0.73–0.86	** < 0.001**	0.80	0.73–0.88	** < 0.001**
Sbp		17.3	1.21	1.15–1.27	** < 0.001**	1.18	1.11–1.25	** < 0.001**	1.19	1.11–1.28	** < 0.001**	1.20	1.11–1.29	** < 0.001**
Dbp		11.1	1.14	1.08–1.20	** < 0.001**	NA	NA	NA	NA	NA	NA	NA	NA	NA
Triglyceride		59.6	1.04	0.99–1.09	0.124									
HDL cholesterol		16.5	0.95	0.91–1.00	0.064									
LDL cholesterol		31.2	0.65	0.62–0.69	** < 0.001**	0.85	0.79–0.91	** < 0.001**	0.81	0.75–0.88	** < 0.001**	0.81	0.75–0.88	** < 0.001**
eGFR		15.6	0.98	0.93–1.03	0.344									
Serum creatinine		0.2	1.41	1.35–1.47	** < 0.001**	NA	NA	NA	1.25	1.17–1.34	** < 0.001**	1.25	1.17–1.34	** < 0.001**
AST		6.7	1.21	1.16–1.26	** < 0.001**	0.98	0.92–1.05	0.613	1.01	0.95–1.08	0.682	0.96	0.89–1.03	0.270
ALT		9.4	0.92	0.88–0.98	0.005									
γ-GTP		19.6	1.41	1.36–1.46	** < 0.001**	1.19	1.13–1.25	** < 0.001**	NA	NA	NA	1.10	1.03–1.17	0.006
Fasting blood glucose		14.9	1.15	1.09–1.22	** < 0.001**	NA	NA	NA	NA	NA	NA	NA	NA	NA
HbA1c		0.5	0.90	0.85–0.95	** < 0.001**	0.94	0.89–1.00	0.068	1.00	0.93–1.07	0.935	0.99	0.92–1.07	0.826
Hematocrit		4.1	1.17	1.10–1.24	** < 0.001**	NA	NA	NA	NA	NA	NA	0.87	0.80-0.94	0.001
Hemoglobin		1.5	1.23	1.16–1.30	** < 0.001**	NA	NA	NA	0.94	0.87–1.02	0.160	NA	NA	NA
RBC		46.1	0.84	0.79–0.89	** < 0.001**	0.79	0.74–0.85	** < 0.001**	NA	NA	NA	NA	NA	NA
MCV		5.1	1.88	1.79–1.99	** < 0.001**	1.31	1.22–1.40	** < 0.001**	1.48	1.38–1.59	** < 0.001**	1.51	1.40–1.63	** < 0.001**
MCH		1.8	2.07	1.97–2.18	** < 0.001**	NA	NA	NA	NA	NA	NA	NA	NA	NA
MCHC		1.1	1.23	1.16–1.30	** < 0.001**	1.01	0.95–1.08	0.718	NA	NA	NA	1.09	1.01–1.18	0.029
Uric acid		1.3	1.45	1.38–1.52	** < 0.001**	NA	NA	NA	NA	NA	NA	NA	NA	NA

The areas marked “NA” in each multivariable model represent factors that were statistically significant in the univariable model but were not applied to the corresponding multivariable model. BMI, body mass index; SBP, systolic blood pressure; DBP, diastolic blood pressure; RBC, red blood cell count; γ-GTP, γ-glutamyltranspeptidase; AST, aspartate; MCH, mean corpuscular hemoglobin; MCHC, mean corpuscular hemoglobin concentration; MCV, mean corpuscular volume; LDL, low density lipoprotein cholesterol; aminotransferase; ALT, alanine transaminase; HDH, high density lipoprotein cholesterol; eGFR, estimated glomerular filtration rate; HR, hazard ratio; CI, confidence interval; NA, not applicable

To verify the proportional hazards assumption, the cumulative incidence of esophageal cancer was evaluated. The hazard ratios among the four quartile-based MCV categories were consistent over the observation period, confirming that the proportional hazards assumption was satisfied (S2 Fig). Even when MCV was categorized, all three multivariable analyses demonstrated that MCV was a risk factor for the development of esophageal cancer (S1 Table and S3 Table).

### Cumulative Incidence of Esophageal Cancer by MCV Quartile Category

**Fig 3:**
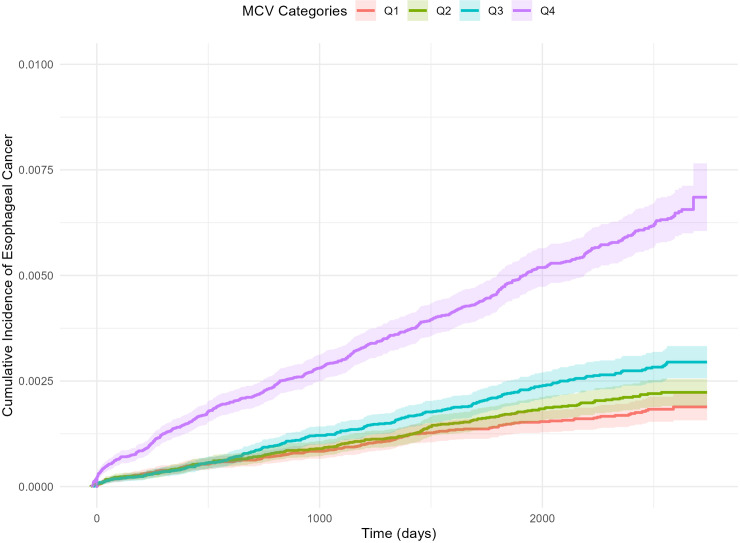
Cumulative incidence of esophageal cancer by MCV quartile category considering death as a competing risk. Q, quartile.

To accurately determine the cumulative incidence of esophageal cancer, incidence was evaluated by MCV quartile category considering death as a competing risk. As shown in Fig 3, the cumulative incidence of esophageal cancer increased with higher MCV levels.

### MCV cutoff value

**Fig 4:**
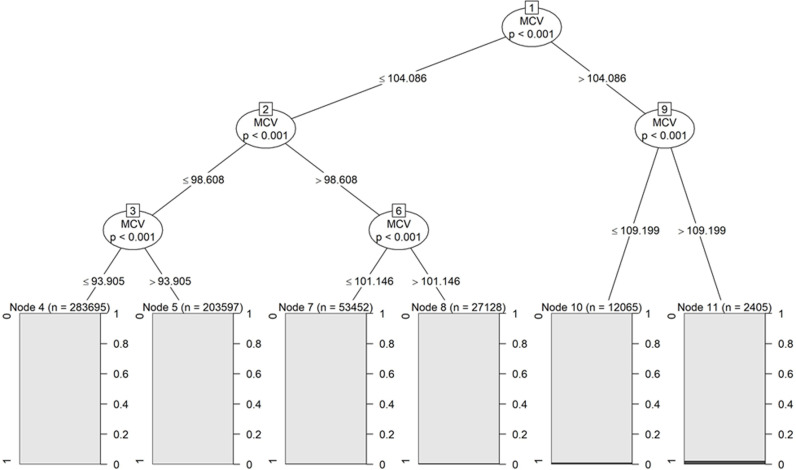
Conditional inference tree for predicting esophageal cancer onset with cutoff values for mean corpuscular volume (MCV).

The cutoff value for predicting esophageal cancer onset using conditional inference tree analysis was 104.086 fl (Fig 4).

### MCV in participants with alcohol use disorder

MCV in participants with alcohol use disorder was compared between those diagnosed with esophageal cancer and the others. Those diagnosed with esophageal cancer had higher MCV than the others (p = 0.006) (Table 3).

**Table 3:** Mean corpuscular volume in participants with alcohol use disorder.

Variable	Participants with comorbid alcohol use disorder		p-value
	With esophageal cancer	Without esophageal cancer	
	(n = 34)	(n = 2354)	
MCV, Mean (SD)	100 (6.26)	96.5 (5.79)	0.006

MCV, mean corpuscular volume; SD, standard deviation

## Discussion

This study is the first to use a large-scale dataset to demonstrate that MCV predicts esophageal cancer onset.

Drinking alcohol and smoking are well-established risk factors for esophageal cancer [3], and it is well known that drinking and smoking act synergistically to increase risk, especially in squamous cell carcinoma [21]. Ethanol absorbed by the body through alcohol consumption is broken down into acetaldehyde by alcohol dehydrogenase. In 2009, the World Health Organization International Agency for Research on Cancer classified “acetaldehyde associated with alcohol consumption” as a carcinogen [22]. Aldehyde dehydrogenase 2 (ALDH2) is an enzyme that metabolizes acetaldehyde, a carcinogen, into acetic acid. Genetic polymorphisms in East Asia result in a high percentage of variants with reduced ALDH2 activity [23]. In individuals with wild-type ALDH2 * 1/ * 1 that provides high enzyme activity, acetaldehyde does not accumulate excessively, and large amounts of alcohol can be consumed; in those with the * 1/ * 2 mutation that provides low enzyme activity, alcohol can be consumed despite facial flushing; and those with the * 2/ * 2 mutation that provides deficient enzyme activity are susceptible to facial flushing, tachycardia, and nausea after even very small amounts of consumption [24]. About 40% of Japanese people have the * 1/ * 2 mutation, in which aldehydes tend to accumulate, and the average amount of alcohol consumed by men is reported to be about 70% of those with * 1/ * 1 [25,26]. Individuals with * 1/ * 2 have the highest risk of cancer due to alcohol consumption, compared to those with * 2/ * 2 who rarely drink [24,27]. Epidemiological studies of esophageal cancer in Japan and Taiwan have shown that the risk of esophageal cancer is higher in drinkers with * 1/ * 2 than in those with * 1/ * 1 [28,29]. Furthermore, Hashimoto et al. reported that MCV was significantly increased in drinkers with * 1/ * 2 compared to those with * 1/ * 1, suggesting that this difference may be due to increased blood aldehyde levels [30]. It is possible that the group diagnosed with esophageal cancer in the present study included more participants with the * 1/ * 2 genotype, which is associated with a higher risk of esophageal cancer onset, and thus had a higher MCV than other participants. Based on the above findings, it is reasonable to hypothesize that increased MCV is a predictor of esophageal cancer onset.

Yokoyama et al. reported that individuals with the * 1/ * 2 genotype had higher MCV than those with * 1/ * 1 when they drank more than moderate amounts of alcohol, but there was no difference when they drank small amounts [31]. In our study, among participants with alcohol use disorder, individuals diagnosed with esophageal cancer had higher MCV than other individuals. This result suggests that the main group in this study diagnosed with esophageal cancer may have included a higher percentage of individuals with the * 1/ * 2 genotype than the others who were not diagnosed with the disease.

Cigarette smoke also contains acetaldehyde [32], and smoking has been reported to increase MCV [33]. Furthermore, like drinking, smoking has also been reported to increase the risk of esophageal cancer synergistically in individuals with the * 1/ * 2 genotype [28]. The details of the mechanism by which drinking and smoking increase MCV are not clear, but it is likely that the same mechanism is responsible for the macrocytosis related to each factor.

The cutoff value for predicting esophageal cancer onset in our study was 104.086 fl. Yokoyama et al. previously reported that macrocytosis with MCV ≥  106 fl was associated with an increased risk of esophageal cancer onset [8,10], and our results did not differ significantly from those in their reports.

In addition to MCV, smoking, amount of alcohol consumption, sex, and low BMI were predictors of esophageal cancer onset in our study, similar to previous reports [34,35]. If smoking and alcohol consumption status can be known in accurate detail, these factors may be extremely useful as predictors of esophageal cancer onset. However, as patients’ self-reports of smoking and alcohol consumption are not always correct, MCV may be a useful predictor in situations where alcohol consumption and smoking are not accurately known.

Hypertension, systolic blood pressure, and low-density lipoprotein (LDL) cholesterol were also independent predictors of esophageal cancer onset in the three multivariable analyses in this study. Amamoto et al. reported no direct causal relationship between hypertension and ALDH2 genotype, although systolic blood pressure and γ-GTP did increase with alcohol consumption [25]. On the other hand, Sasakabe et al. reported that LDL cholesterol per 10 g/day of alcohol consumption was significantly lower in Japanese men with the * 1/ * 2 and * 2/ * 2 genotypes than in those with the * 1/ * 1 genotype [36]. Participants diagnosed with esophageal cancer in our study had lower LDL cholesterol than the others, suggesting that a higher percentage of participants with the * 1/ * 2 genotype may have been present in those diagnosed with the disease.

For the variables examined separately in the three multivariable models, red blood cell count and hematocrit level were independent predictors of esophageal cancer onset. Despite the lower red blood cell counts in individuals diagnosed with esophageal cancer than in the others, hemoglobin and hematocrit levels were conversely higher in those diagnosed with the disease. It has been reported that moderate or heavy drinking is associated with lower red blood cell counts in individuals with the * 1/ * 2 genotype than in those with * 1/ * 1, but is not associated with differences in hemoglobin and hematocrit levels [31]. Jean et al. reported that smoking was associated with increased hemoglobin and hematocrit levels; however, they also reported that red blood cell count was not significantly associated with alcohol consumption and smoking [33]. In the present study, smoking may have played a role in the elevated hemoglobin and hematocrit levels in individuals diagnosed with esophageal cancer.

The above results suggest that it may be appropriate to carry out endoscopic examination for esophageal cancer when MCV is greater than 104 fl. Early detection before symptoms appear is essential for improving prognosis, and MCV, which can conveniently be identified by blood sampling alone, may be a useful indicator to perform endoscopy.

The demographic group that these findings may be most relevant to is individuals of East Asian descent. While most Caucasians and individuals of African descent have the ALDH2 * 1/ * 1 genotype [37], Li et al. reported that the ALDH2 * 1/ * 2 and * 2/ * 2 genotypes occur most frequently in Southeast China and also with high frequency in countries such as Japan, Korea, and Mongolia [38]. Thus, MCV is likely to be most valuable as predictive factor for the onset of esophageal cancer in East Asian countries where the frequency of ALDH2 polymorphisms is similar to that of Japanese.

These findings also suggest an intriguing wider implication: in addition to MCV being a predictor of esophageal cancer, it may also be a predictor of other cancers for which drinking and smoking are risk factors. In particular, the oral cavity, pharynx, and larynx are located close to the esophagus, and alcohol consumption and smoking are also well-known risk factors for these cancers [39,40]. Therefore, MCV is likely to be useful for predicting their onset as well.

This study has several limitations. First, its findings are based on data from a specific region in Japan, limiting generalizability to other populations. Second, we do not know the exact amount of alcohol consumption or smoking among those who underwent health checkups. Some of this data was self-reported, and therefore may have been affected by recall and social desirability biases, potentially affecting the accuracy of the alcohol and smoking consumption records. This in turn may have resulted in under- or overestimation of the associations between alcohol, smoking, and MCV. Third, individuals who have stopped drinking or smoking for an extended period may still be at risk of developing esophageal cancer, but their MCV may not be increased. Fourth, it has been reported that the risk of esophageal cancer decreases with vegetable and fruit intake, even among drinkers and smokers [41], but the SKDB had insufficient information on food intake for the present study to assess this factor. Fifth, the health checkup data did not include information on the ALDH2 gene. The impact of ALDH2 polymorphism should be addressed in future research. Sixth, the codes used in this study did not distinguish between squamous cell carcinoma and adenocarcinoma, and stages of esophageal cancer progression were not included in the database. As MCV is associated with the development of esophageal cancer rather than with its progression, its correlation with stage of esophageal cancer is expected to be limited. However, in cases of advanced esophageal cancer, we cannot ruled out the potential impact of tumor bleeding and malnutrition on hemoglobin levels and MCV due to impaired oral intake. Finally, the lack of sensitivity analysis for missing data may impact the reliability of the results.

In conclusion, MCV, a well-known indicator of alcohol and tobacco consumption, is a valuable marker for predicting esophageal cancer onset. It may be appropriate to carry out endoscopic examination when MCV is greater than 104 fl, especially in individuals of East Asian descent.

## Supporting information

S1 Table Spearman correlation coefficients for significant factors in univariate Cox regression analysis. Bold type indicates a correlation of >  0.4. BMI, body mass index; SBP, systolic blood pressure; DBP, diastolic blood pressure; RBC, red blood cell count; LDL, low density lipoprotein cholesterol; AST, aspartate aminotransferase; γ-GTP, γ-glutamyltranspeptidase; RBC, red blood cell count; MCH, mean corpuscular hemoglobin; MCV, mean corpuscular volume; MCHC, mean corpuscular hemoglobin concentration; NA, not applicable.
(XLSX)

S2 Table Univariable and multivariable Cox regression analysis of esophageal cancer onset by MCV category. The areas marked as “NA” in each multivariable model represent factors that were statistically significant in the univariable model but were not applied to the corresponding multivariable model. BMI, body mass index; SBP, systolic blood pressure; DBP, diastolic blood pressure; RBC, red blood cell count; γ-GTP, γ-glutamyltranspeptidase; AST, aspartate; MCH, mean corpuscular hemoglobin; MCHC, mean corpuscular hemoglobin concentration; MCV, mean corpuscular volume; LDL, low density lipoprotein cholesterol; aminotransferase; ALT, alanine transaminase; HDH, high density lipoprotein cholesterol; eGFR, estimated glomerular filtration rate; HR, hazard ratio; CI, confidence interval; NA, not applicable, Q, quartile.
(TIF)

S1 Fig Cumulative Incidence of Esophageal Cancer by MCV Quartile Category. Q, quartile.
(XLSX)
