# Correction: High mean corpuscular volume as a predictor of esophageal cancer: A cohort study based on the Japanese Shizuoka Kokuho Database

**DOI:** 10.1371/journal.pone.0330046

**Published:** 2025-08-08

**Authors:** The PLOS ONE Staff

## Notice of Republication

The author order was incorrect. The correct order is:

Shinsuke Sato, Emi Ohata, Eiji Nakatani, Philip Hawke, Hatoko Sasaki, Erina Nagai, Yusuke Taki, Masato Nishida, Masaya Watanabe, Ko Ohata, Hideyuki Kanemoto, Akira Sugawara

## Supporting information

S1 File Originally published, uncorrected article. (PDF)

S2 File Republished, corrected article. (PDF)
