# Supplementary figures and images for: High mean corpuscular volume as a predictor of esophageal cancer: A cohort study based on the Japanese Shizuoka Kokuho Database
Source: PLoS One. 2025 Feb 11;20(2):e0318791. doi: 10.1371/journal.pone.0318791 (PMC11813134; doi:10.1371/journal.pone.0318791)

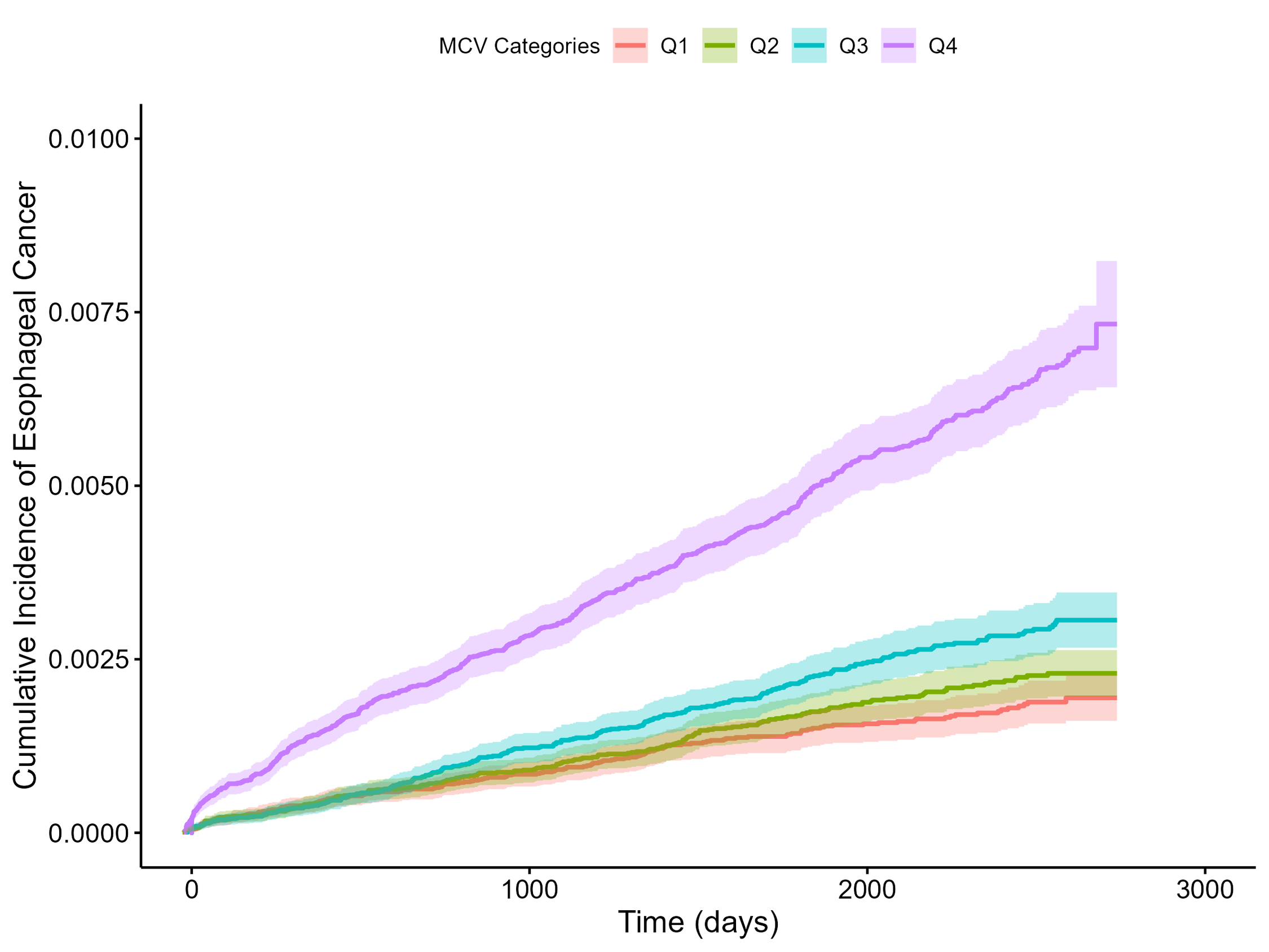

Supplement: S2 Table — The areas marked as “NA” in each multivariable model represent factors that were statistically significant in the univariable model but were not applied to the corresponding multivariable model. BMI, body mass index; SBP, systolic blood pressure; DBP, diastolic blood pressure; RBC, red blood cell count; γ-GTP, γ-glutamyltranspeptidase; AST, aspartate; MCH, mean corpuscular hemoglobin; MCHC, mean corpuscular hemoglobin concentration; MCV, mean corpuscular volume; LDL, low density lipoprotein cholesterol; aminotransferase; ALT, alanine transaminase; HDH, high density lipoprotein cholesterol; eGFR, estimated glomerular filtration rate; HR, hazard ratio; CI, confidence interval; NA, not applicable, Q, quartile. (TIF) [file pone.0318791.s002.tif]
